# miR‐1322 regulates ChREBP expression via binding a 3′‐UTR variant (rs1051943)

**DOI:** 10.1111/jcmm.13805

**Published:** 2018-08-05

**Authors:** Ying Zhang, Sen‐Lin Hu, Dong Hu, Jian‐Gang Jiang, Guang‐Lin Cui, Xing‐De Liu, Dao Wen Wang

**Affiliations:** ^1^ Division of Cardiology Tongji Hospital Tongji Medical College Institute of Hypertension and Department of Internal Medicine Hubei Province Key Laboratory of Genetics and Molecular Mechanisms of Cardiological Disorders Huazhong University of Science and Technology Wuhan China; ^2^ Department of Cardiology Affiliated Hospital of Guizhou Medical University Guiyang China

**Keywords:** 3′‐UTR, ChREBP, lipid, metabolism, miR‐1322

## Abstract

The carbohydrate response element‐binding protein (ChREBP), also referred to as MLXIPL, plays a crucial role in the regulation of glucose and lipid metabolism. Existing studies have shown an association between genetic variations of the ChREBP gene and lipid levels, such as triglycerides and high‐density lipoprotein cholesterol. However, mechanistic studies of this association are limited. In this study, bioinformatic analysis revealed that the polymorphism rs1051943A occurs in the complementary binding sequence of miR‐1322 in the ChREBP 3′‐untranslated region (UTR). Studies of potential mechanisms showed that the A allele could facilitate miR‐1322 binding, and luciferase activity significantly decreased when co‐transfected with a ChREBP 3′‐UTR luciferase reporter vector and miR‐1322 mimics in HepG2 cells. Furthermore, miR‐1322 significantly regulated the expression of ChREBP downstream genes and reduced the synthesis of lipids. The expression of miR‐1322 was up‐regulated by glucose and palmitic acid stimulation. Population studies showed that rs1051943‐A allele was only found in the Han Chinese and Uighur ethnic groups, different from European populations (G allele frequency = 0.07). In summary, we provide evidence that the rs1051943 A allele creates a functional miR‐1322 binding site in ChREBP 3′‐UTR and post‐transcriptionally down‐regulates its expression, possibly associated with levels of plasma lipids and glucose.

## 1 INTRODUCTION

Metabolic syndrome (MetS) is a worldwide public health issue and a challenging clinical problem.^1^ Growing mortality and disability could attribute to MetS‐related diseases, such as stroke and myocardial infarction.^2^ To date, many studies have established that dyslipidaemia and hyperglycaemia are main risk factors for metabolic syndrome.^3^ ChREBP, firstly discovered by Spanish scientists as WBSCR14,^4^ plays an important role in glucose and lipid homoeostasis.^5^ Yamashita et al found that ChREBP could bind to the promoter region of the L‐type pyruvate kinase (LPK) gene.^6^ It is now well established that ChREBP is a key transcription factor involved in glycolysis and lipogenesis.^7^

Considering the important roles of ChREBP in glucose and lipid metabolism, whether genetic factors are involved in the gene expression is highly concerned. Many single‐nucleotide polymorphisms (SNPs) in or near the ChREBP gene have been found to be associated with plasma triglyceride levels by genome‐wide association studies (GWASs).^8^, ^9^, ^10^ During the last decade, more than 20 studies confirmed the association between ChREBP gene variations and plasma triglyceride levels, as well as the association with coronary artery disease (CAD).^11^, ^12^, ^13^, ^14^, ^15^, ^16^

MicroRNAs (miRNAs) are small non‐coding RNAs (19‐22 nucleotides) known to be regulators of gene expression at post‐transcriptional levels.^17^ The previous studies have shown that miRNAs could alter gene expression and individual susceptibility to disease traits, and variants located in these miRNA binding sites may alter clinical characteristics and disease susceptibility.^18^, ^19^, ^20^ To our knowledge, whether polymorphism in the 3′‐UTR of ChREBP could modify gene expression and further influence triglycerides and glucose levels has not been clearly demonstrated. Therefore, we performed a bioinformatics analysis to identify the potential functional variant in the 3′‐UTR of ChREBP. As a result, the known polymorphism, rs1051943, was found in the seed binding site of miR‐1322, one of the 69 miRNAs previously identified to be associated with abnormal lipid levels.^21^ Based on these results, we hypothesized that the human rs1051943 polymorphism could regulate ChREBP expression by influencing miR‐1322 binding and thus affect plasma levels of lipids and glucose.

## 2 MATERIALS AND METHODS

### 2.1 Bioinformatics analysis

Bioinformatics analysis was performed with three distinct software tools. miRanda (http://www.microrna.org/), RegRNA (http://www.lncrnablog.com/) and miRTarBase (http://mirtarbase.mbc.nctu.edu.tw/) were used to predict miRNA binding sites in 3′‐UTR of ChREBP. The results were then confirmed by TargetScan 7.1 (http://www.targetscan.org/). With data from Asia, Europe, Africa and China, the 1000 Genomes Project (http://www.hapmap.org/, phase3) provided the SNPs in 3′‐UTR of ChREBP and the minor allele frequency (MAF), which was greater than 0.01.

### 2.2 Luciferase assay

The 671‐bp ChREBP 3′‐UTR sequence flanking rs1051943 containing a wild‐type allele (rs1051943‐A) was amplified from human DNA by polymerase chain reaction (PCR), with Mlu1 or HindIII restriction enzyme cutting site at 5′ and 3′ end, respectively. PCR products were then digested and inserted into a pMIR luciferase reporter vector (Beijing AuGCT Biotechnology Co., Ltd, China). A mutated vector containing ChREBP 3′‐UTR sequence was generated by overlap PCR using primers carrying mutated alleles (forward primer: 5′‐TCCTGGCCAGACCCTGCT‐3′, reverse primer: 5′‐AACCAGCAGACAGTTTTTGC ‐3′). HEK293T and HepG2 cells (1 × 10^^6^ cells per well) in 24‐well plates were co‐transfected with 400 ng of pMIR‐A (rs1051943‐A) or pMIR‐G (rs1051943‐G) plasmid, 50 ng of Renilla luciferase plasmid, 100 nmol of miR‐1322 mimics or mimics negative control (RIBOBIO Co., Ltd, Guangzhou, China) and 200 nmol of miR‐1322 inhibitor or inhibitor negative control (RIBOBIO Co., Ltd, Guangzhou, China), using Lipofectamine 2000 (Invitrogen, Carlsbad, CA, USA). Cy3‐labelled transfection control (RIBOBIO Co., Ltd, Guangzhou, China) was used to detect the infection efficiency, as shown in Figure S1. The sequences of miRNA‐1322 mimics and inhibitors are shown in Table S1. Cells were incubated at 37°C and 5% CO_2_. After 48 hours, the cells were washed using cold phosphate‐buffered saline (PBS) and lysed with passive lysis buffer (Promega, WI, USA). Luciferase activities were then measured by a luminometer (SIRIUS, Pforzheim, Germany) according to the manufacturer's instructions. Luciferase expression levels were adjusted with reference to Renilla luciferase activity. Six independent experiments were performed for each reporter vector. Mouse ChREBP 3′‐UTR was constructed into the pMIR‐Report vector using the above‐mentioned procedure (forward primer: 5′‐TCCTGGTGAAAGTTTCCAAGC‐3′, reverse primer: 5′‐CCCAAGTACTGGGATTAAAGGTG‐3′). Vectors were then co‐transfected with hsa‐miR‐1322 mimic (100 nmol/L) or mimic negative control (100 nmol/L) in HEK293T cells.

### 2.3 Recruitment for hypertriglyceride (HTG) patients and control participants

The sequencing cohort included 169 HTG individuals and 313 control participants of Han Chinese descendant. The individuals with HTG were unrelated family members diagnosed with Fredrickson hypertriglyceridemia phenotype,^22^ defined as having fasting plasma TG >10 mmol/L from a single tertiary referral lipid clinic. Patients underwent a complete medical history and examination; basic clinical, biochemical and demographic variables were collected. Ethnically and geographically matched controls were collected from individuals undergoing routine health examinations at Tongji Hospital in Wuhan, Hubei province. We chose controls with maximum recorded fasting plasma triglyceride concentrations <2.3 mmol/L to exclude undiagnosed HTG. No controls had disease conditions or morbid obesity (body mass index > 30 kg/m^2^), and there was no use of medication among these healthy control participants. None of the members were using lipid‐lowering medication when the blood sample was taken. In fasting venous blood samples, we measured total cholesterol, HDL‐C, TG and LDL‐C on the Rocha modular DPP system according to standard procedures at the Department of Clinical Chemistry, Tongji Hospital. The clinical characteristics of the samples are shown in Table 1. Additionally, 96 of the Chinese ethnic Uighur participants were also included in the sequencing cohort (Table S2). The Institutional Review Board at Tongji Hospital approved this study. Written informed consent was obtained from all three participants. Genetic experiments were conducted according to the principles expressed in the Declaration of Helsinki.

**Table 1:** Baseline characteristics of the study samples

Characteristics	Chinese Han	
	Control (n = 313)	HTG (n = 169)
Age, years	59.8 ± 10.9	47.2 ± 12.6
Men, %	46.0	74.5
SBP, mm Hg	123.5 ± 17.9	121.3 ± 19.3
DBP, mm Hg	67.0 ± 10.3	68.6 ± 10.3
BMI, kg/m2	22.20 ± 3.19	25.38 ± 3.19
TG, mmol/L	0.88 ± 0.48	13.70 ± 4.34
TC, mmol/L	4.70 ± 0.95	6.78 ± 2.36
HDL, mmol/L	1.65 ± 0.40	0.85 ± 0.21
LDL, mmol/L	2.52 ± 0.8	1.87 ± 0.77
Hypertension	0	0
Type 2 diabetes	0	0

SBP, systolic blood pressure; DBP, diastolic blood pressure; BMI, body mass index.

### 2.4 Genetic variation screening

In order to identify existing variants in the ChREBP gene, Sanger sequencing was performed. Details on sample sequencing were described in our previous report.^20^ Briefly, PCR fragments covering the 3′‐UTR of ChREBP (ChREBP consensus sequence, NC_000007.13, GRCh37.p13) were generated using primers (forward primer: 5′‐AGCTGGGCACATCTACCAGTAT‐3′ and reverse primer: 5′‐CACTGCCAACAGGCTCTCTCT‐3′). Applied Biosystems 3130xl capillary sequencer (Applied Biosystems, Foster City, CA) was used to analyse fluorescent dye‐terminator cycle products from the PCR fragments. Putative polymorphisms were identified through the Chromas program (Technelysium Pty. Ltd., Helensvale, Queensland, Australia). Results were then confirmed by two independent observers. All identified variants were confirmed by repeat sequencing.

### 2.5 Western blots

Cells grown in 6‐well plates were transfected with miR‐1322 (100 nmol/L), miRNA mimic negative control (100 nmol/L), hsa‐miR‐1322 inhibitor (200 nmol/L) or miRNA inhibitor control (200 nm), using Lipofectamine 2000 (Invitrogen, Carlsbad, CA, USA) according to the manufacturer's instructions. After 48 hours, cells were disposed to radio immunoprecipitation assay lysis buffer (Beyotime Biotechnology, Beijing, China) and incubated at 4°C for 20 minutes. The supernatant was collected after centrifuging at 12,000 *g* at 4°C for 20 minutes. Protein concentration was determined by BCA protein assay kit (Boster Biological Technology, Wuhan, China). Cell lysates were separated by 8% SDS–polyacrylamide gel electrophoresis and transferred to polyvinylidene difluoride (PVDF) membranes. After 2 hours blocking with 5% non‐fat milk at room temperature, the ChREBP antibody (Abcam, Boston, USA) was utilized to perform immunoblots at 4°C, followed by incubation with a peroxidase‐conjugated secondary antibody. The bands were visualized by enhanced chemiluminescence reagents (Pierce Chemical, Rockford, IL) and quantified by densitometry. Other immunoblotting was achieved using the antibodies, PKLR (Boster Biological Technology, Wuhan, China), FASN (Boster Biological Technology, Wuhan, China) and ACC (Boster Biological Technology, Wuhan, China).

### 2.6 Triglycerides and total cholesterol assays

HepG2 cells in 6‐well plates were transfected with hsa‐miR‐1322 (100 nmol/L), miRNA mimic negative control (100 nmol/L), hsa‐miR‐1322 inhibitor (200 nmol/L) or miRNA inhibitor control (200 nm). After transfection for 24 hours, cell lysates were extracted as described previously.^20^ Triglyceride and total cholesterol levels were determined using a commercial assay kit (Nanjing Jiancheng Bioengineering Institute, China) normalized to protein concentration following the manufacturer's instructions. The assay sensitivity was 0.01 mmol/mL, and average intra‐ and inter‐assay coefficients of variation were 3% and 5%, respectively. The absorbance was measured using a BioStack Microplate Stacker (BioTek, WI, USA) according to the instruction manual.

### 2.7 Quantitative real‐time PCR

Total RNA was extracted from human normal tissues including liver, adipose tissue, large intestine, small intestine, heart, lung and cell lines including HepG2, 7721, Hep3B, Hep1, L02 and 293T, using a Trizol Reagent Kit (Invitrogen, Carlsbad, CA) according to the manufacturer's instructions. Tissue samples were obtained from normal distal tissue of tumour patients as previously described.^20^ The miR‐1322 expression level was quantified by real‐time quantitative PCR using power SYBR green PCR master mix (Applied Biosystems Inc.). The small nuclear RNA U6 (RIBOBIO Co., Ltd, Guangzhou, China) was employed as the internal reference. All real‐time reactions were run in triplicate using the ABI 7900 fast real‐time PCR system (Applied Biosystems Inc.). The tissue sample studies were approved by the Institutional Review Board of Tongji Hospital and Tongji Medical College conformed to the Declaration of Helsinki.

HepG2 cells were seeded in 6‐well plates. After adherence for 24 hours, cells were transfected with hsa‐miR‐1322 (100 nmol/L), miRNA mimic negative control (100 nmol/L), hsa‐miR‐1322 inhibitor (200 nmol/L) or miRNA inhibitor control (200 nm). After 24 hours, cells were harvested, and total RNA was extracted using a Trizol Reagent Kit (Invitrogen, Carlsbad, CA) according to the manufacturer's instructions. One microgram of total RNA was reverse‐transcribed using PrimeScript™ RT reagent kit with gDNA eraser (TakaraBio, Otsu, Japan). The mRNA levels were quantified by real‐time quantitative PCR using power SYBR green PCR master mix (Applied Biosystems Inc.), with 18S rRNA employed as internal reference. All real‐time reactions were run in triplicate using the ABI 7900 Fast Real‐Time PCR System (Applied Biosystems Inc.). The primers used are listed in Table S3.

### 2.8 Vector construction and miRNA sequencing

cDNA from C57BL/6J mouse liver was amplified using hsa‐miR‐1322‐specific primers (RIBOBIO Co., Ltd, Guangzhou, China). PCR products were then TA‐cloned into pMD 19‐T vector (Code No. 6013) following the manufacturer's instructions (TakaraBio, Otsu, Japan). Positive clones were selected and sequenced using universal primers. The sequence of miR‐1322 was found in chromosome 19 (GRCm38.p4, 29842645 to 29842663) using the UCSC genome browser (http://genome.ucsc.edu/). PCR product of approximately 600 bp flanking the potential miR‐1322 locus was amplified from the extracted DNA of C57BL/6J mice (forward primer: 5′‐GGCAGAGGCAGGTGGATT‐3′, reverse primer: 5′‐TGTTGGGATTACGGTGCCT‐3′). Products were re‐sequenced using forward primer.

### 2.9 Statistical analysis

All statistical analyses were performed with SPSS 17.0 (SPSS Inc, Chicago, III) for Windows (Microsoft Corp, Redmond, WA, USA). Linkage disequilibrium (LD) was calculated using Haploview version 4.1. The chi‐squared test was used to assess deviations of genotype frequency from the Hardy‐Weinberg assumption. Quantitative variables were expressed as mean ± standard error of the mean (SEM) of n experiments. Comparisons of quantitative variables between two groups were performed by the paired or unpaired Student's *t* test. One‐way ANOVA was used to compare multiple variables followed by post hoc *t* tests. All probability values were two‐sided, and *P* < 0.05 was considered to be significant. Risk allele frequency was compared using logistic regression analysis based on the different genetic models adjusted by traditional risk factors in CHD patients

## 3 RESULTS

### 3.1 Bioinformatics analysis of ChREBP 3′‐UTR

**Figure 1:**
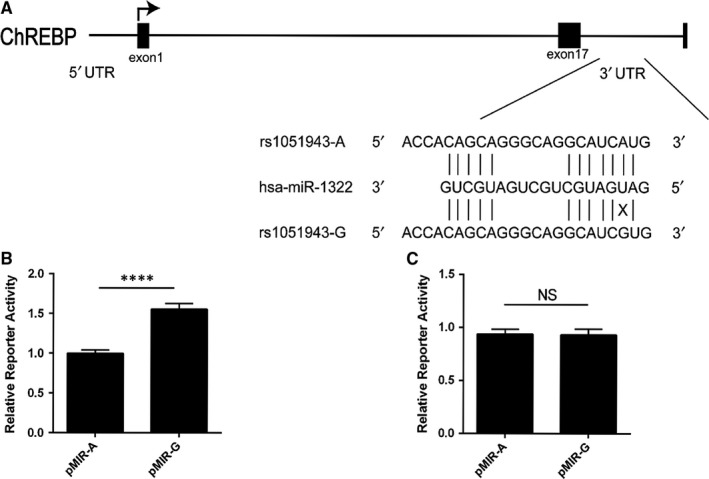
Functional analysis of rs1051943 A/G polymorphism. A, ChREBP gene structure and the rs1051943 polymorphism located in the 3’‐UTR at the miR‐1322 binding site. The rs1051943 polymorphism is a A–G change (mRNA sequence as reference) located in the predicted binding site of miR‐1322. According to Watson‐Crick mode, rs1051943 A allele base‐paired with U allele at the miRNA seed sequence (shown with a vertical line), whereas G allele did not (shown with a cross). B, C, Effects of rs1051943A/G allele on ChREBP 3’‐UTR constructs activity in HepG2 cells (B) and 293T cells (C). ChREBP 3’‐UTR reporter activity is expressed relative to the co‐transfected Renilla luciferase activity. Three independent experiments containing six replicates were performed in each cell line. For each comparison, mutant construct was compared with the wild‐type construct. All values are mean ± SEM of three independent experiments with six replicates. *P* < 0.0001 (****), NS (no significance)

Bioinformatics analysis demonstrated that 30 miRNAs might contain binding sites in 3′‐UTR of ChREBP (Table S4) in which 13 miRNAs are confirmed by TargetScan 7.1 as shown in Table 2. Among these miRNAs, only miR‐1322 was found to be associated with abnormal lipid levels.^21^ The variant rs1051943 is an A to G change (mRNA sequence as reference), and computer alignment demonstrated that this SNP was located in the miR‐1322 binding site in ChREBP 3′‐UTR (Figure 1A).

**Table 2:** List of miRNAs predicted in the ChREBP 3’‐UTR confirmed by distinct software tools

miRNA	ChREBP NM_032951		website
	Start^a^ match	End^a^ match	
hsa‐miR‐6894‐3p	564	585	miRTarBase, TargetScan7.1
hsa‐miR‐4763‐5p	563	586	miRTarBase, TargetScan7.1
**hsa‐miR‐1322**	**569**	**591**	miRTarBase, TargetScan7.1
hsa‐miR‐4643	573	594	miRTarBase, TargetScan7.1
hsa‐miR‐3692‐3p	351	373	miRTarBase, TargetScan7.1
hsa‐miR‐6881‐3p	356	378	miRTarBase, TargetScan7.1
hsa‐miR‐3124‐3p	353	375	miRTarBase, TargetScan7.1
hsa‐miR‐130b‐5p	354	376	miRTarBase, TargetScan7.1
hsa‐miR‐877‐3p	356	378	miRTarBase, TargetScan7.1
hsa‐miR‐5193	358	380	miRTarBase, TargetScan7.1
hsa‐miR‐5196‐3p	361	383	miRTarBase, TargetScan7.1
hsa‐miR‐18a‐3p	362	584	miRTarBase, TargetScan7.1
miR‐4685‐5p	85	108	RegRNA, TargetScan7.1

miRNA with binding site flanking known SNP was indicated as bold.

a Start and end sites were numbered relative to the stop codon based on NCBI GRCh37, NM 032951 as reference.

### 3.2 Effects of polymorphisms on activity of ChREBP 3′‐UTR in vitro

In HepG2 cells, the luciferase reporter activity of the pMIR‐G vector (containing G allele of rs1051943) is significantly increased (58.1 ± 7.3%, *P* < 0.0001) when compared with the pMIR‐A construct (containing A allele of rs1051943) as shown in Figure 1B. No significant difference was found between pMIR‐G and pMIR‐A in HEK293T cells (Figure 1C). These results indicate that endogenous hepatic regulator factor could target ChREBP 3′‐UTR and decrease ChREBP 3′‐UTR luciferase expression.

### 3.3 Rs1051943 of ChREBP occurring in miR‐1322 binding site

**Figure 2:**
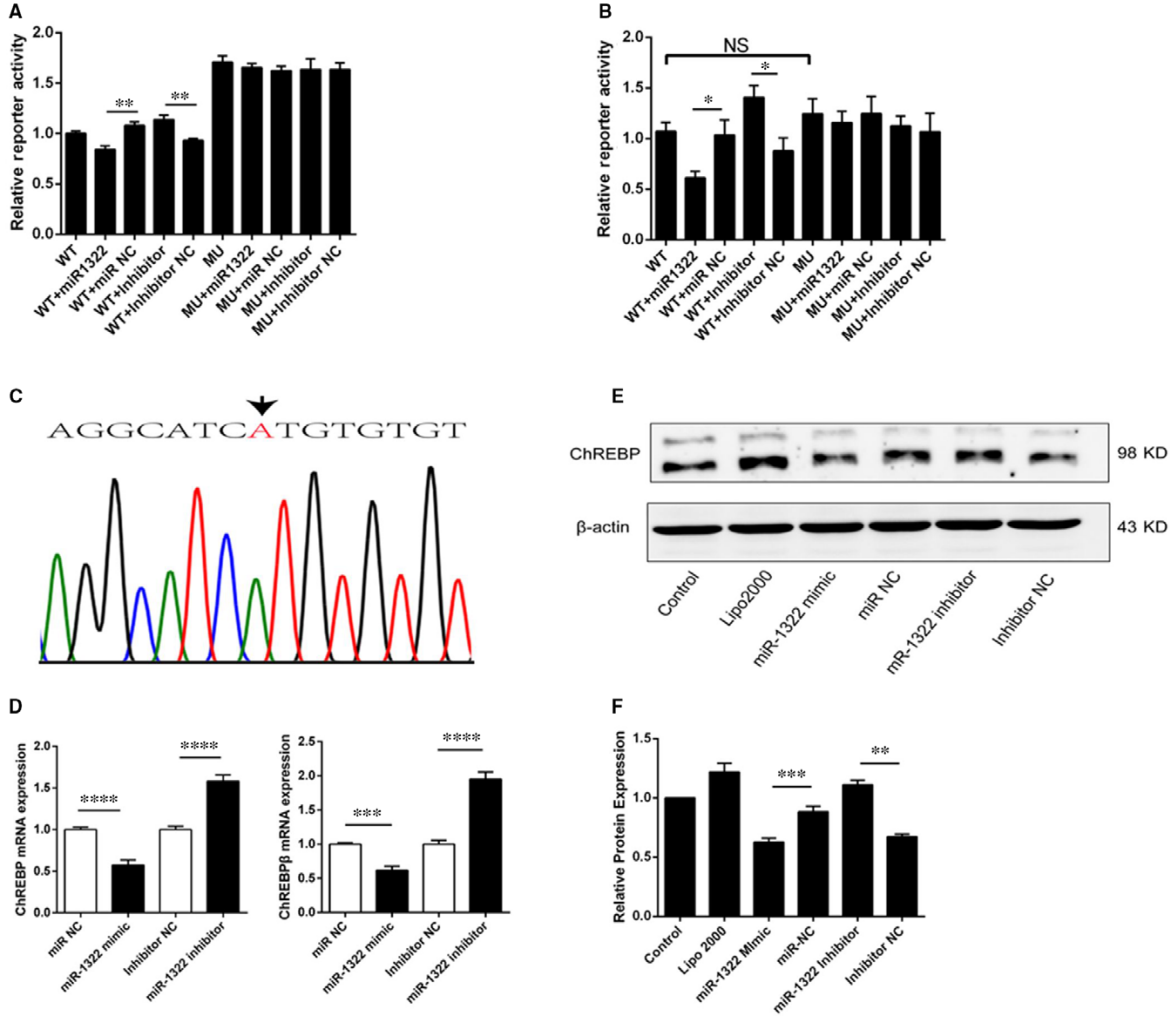
Functional validation of the miR‐1322 binding site in ChREBP 3’‐UTR and the influence of SNP rs1051943. A, B, Interaction between miR‐1322 and ChREBP expression using reporter gene assay in HepG2 cells (A) and 293T cells (B). Plasmid pMIR‐A or pMIR‐G was co‐transfected with miR‐1322 mimics, negative control miRNA (miR NC), miR‐1322 inhibitor or inhibitor negative control (Inhibitor NC). For each transfection, at least six replicates were performed. (C) DNA sequences proximal to rs1051943 in HepG2 cell line. D, E, F, Overexpression of miR‐1322 reduced ChREBP expression through binding to the 3’‐UTR of ChREBP. Inhibition of miR‐1322 increased ChREBP expression in HepG2 cells analysed by real‐time PCR (D) and Western blotting (E, F). Columns, mean of three independent experiments; error bars, SEM. Results were means of triplicate experiments with at least six replicates. *P* < 0.05 (*), *P* < 0.01(**), *P* < 0.001 (***), *P* < 0.0001 (****), NS (no significance)

Given that rs1051943 was located in the conserved region of ChREBP (UCSC Genome Browser; http://genome.ucsc.edu/) and in the miR‐1322 binding site, we focused on rs1051943 for further functional analysis. To test the prediction model that miR‐1322 could functionally interact with 3′‐UTR of ChREBP, HepG2 cells were co‐transfected with pMIR‐A or pMIR‐G, and miR‐1322 mimics or control mimics. Compared with control miRNA, the ChREBP 3′‐UTR construct containing A allele showed a significant reduction in luciferase activity in the presence of miR‐1322 in HepG2 cells (‐24.7 ± 3.4%, *P* = 0.0024) (Figure 2A) and 293T cells (−38.8 ± 6.3%, *P* = 0.021) (Figure 2B). Conversely, no significant changes were observed in the G allele. The inhibition of miR‐1322 expression, using its inhibitor, significantly up‐regulated the luciferase activity of constructs in HepG2 cells (23.8 ± 5.5%, *P* = 0.002) (Figure 2A) and 293T cells (37.1 ± 8.1%, *P* = 0.011) (Figure 2B).

To elucidate the effect of miR‐1322 on endogenous ChREBP expression, five human hepatic cell lines including HepG2, 7721, Hep3B, Hep1 and L02 were re‐sequenced, and all were identified to be AA genotype (Figure 2C, Figure S2). Because the expression of ChREBP was higher in HepG2 cells than in other hepatic cell lines (Figure S3), HepG2 was selected for conducting further experiments. Real‐time PCR and Western blot results showed that the expression of ChREBP was suppressed by miR‐1322; conversely, inhibition of miR‐1322 significantly up‐regulated the expression of ChREBP in HepG2 cells as shown in Figures 2D (mRNA), 2E and 2F (protein). These results confirmed miR‐1322 as a negative transcription regulator of ChREBP in vitro.

### 3.4 The effects of miR‐1322 on glycolysis and lipogenesis in vitro

**Figure 3:**
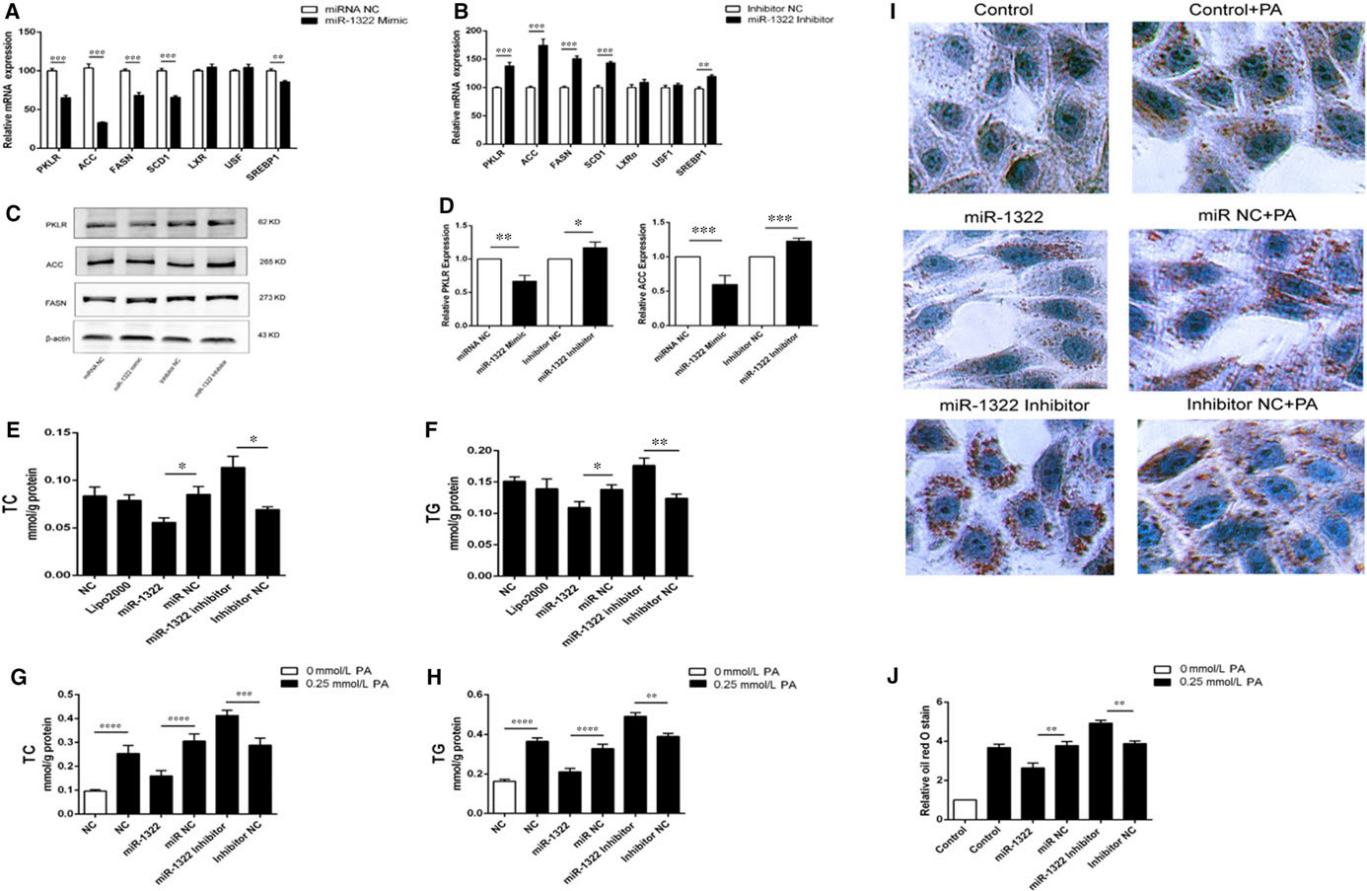
Effect of miR‐1322 in HepG2 cells. Overexpression (A) or inhibition (B) of miR‐1322 alters the mRNA expression of the downstream genes of ChREBP. Western blot analysis and (C) ImageJ quantification of their protein levels (D). Cholesterol (E) and triglyceride (F) levels of HepG2 cell lysis. With the palmitate intervention, cholesterol (G) and triglyceride (H) levels of HepG2 cell lysis and Oil Red O staining analysis of HepG2 cells (I and J). PKLR, pyruvate kinase L/R; ACC, acetyl‐CoA carboxylase; FASN, fatty acid synthase; SCD1, stearoyl‐CoA desaturase 1; LXRα, liver X receptor α; USF1, upstream transcription factor 1; SREBP1, sterol regulatory element‐binding protein 1. *P* < 0.05 (*), *P* < 0.01(**)

Because ChREBP was an important regulator of lipid metabolism, we further explored whether miR‐1322 could regulate the process of glycolysis and lipogenesis. Overexpression of miR‐1322 resulted in decreased expression of PKLR (pyruvate kinase L/R) (−34.8 ± 3.2%, *P* < 0.0001), ACC (acetyl‐coA carboxylase) (−67.1 ± 0.9%, *P* < 0.0001), FASN (fatty acid synthase) (−31.6 ± 3.4%, *P* < 0.0001) and SCD1 (stearoyl‐CoA desaturase 1) (−34.3 ± 2.0%, *P* < 0.0001) as compared with control miRNA in HepG2 cells (Figure 3A), whereas miR‐1322 inhibitor increased the expression of PKLR, ACC, FASN, and SCD1 (Figure 3B). Through miR‐1322 intervention, the expression of LXRα (liver X receptorα) and USF1 (upstream transcription factor 1) showed no significant difference, while SREBP1 (sterol regulatory element‐binding transcription factor 1) expression was modestly decreased (−18.39 ± 2.1%, *P* < 0.0001) (Figure 3A). Similar results were observed in the presence of miR‐1322 inhibitor (Figure 3B). In agreement with the altered mRNA levels, protein levels of PKLR and ACC were increased compared with control group (Figure 3C,D). In addition, miR‐1322 intervention could also regulate the accumulation of cholesterol (Figure 3E) and triglycerides (Figure 3F) in HepG2 cells. In order to better understand the effects of miR‐1322 on lipogenesis in vitro*,* the palmitate‐treated HepG2 cells were used. Palmitate‐induced lipid accumulation was attenuated by miR‐1322, as shown in Figure 3G (cholesterol) and 3H (triglycerides). The Oil Red O staining showed similar results (Figure 3I and 3J).

### 3.5 The effects of miR‐1322 on insulin resistance

**Figure 4:**
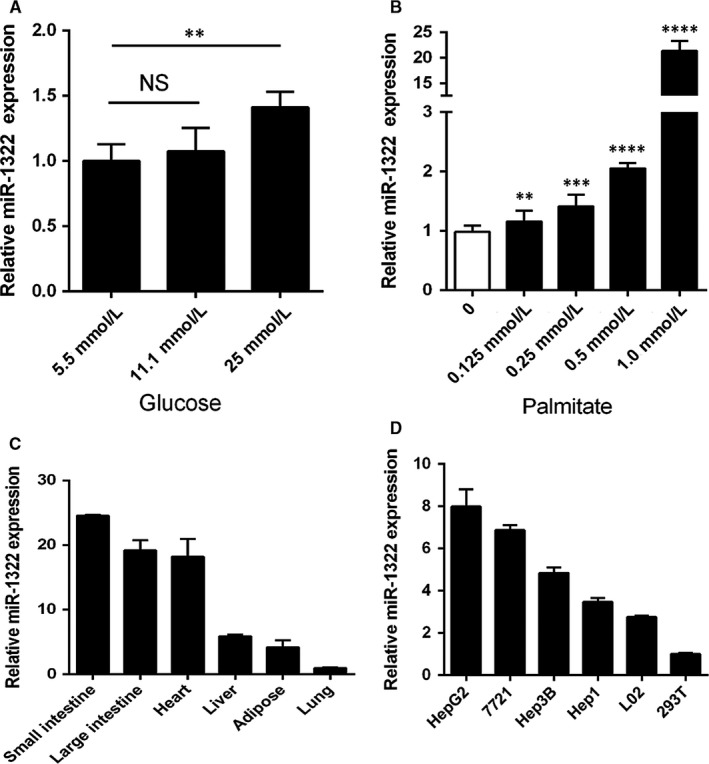
Expression of miR‐1322 in cell model for insulin resistance induced by palmitate or high glucose cell culture media. A, B, HepG2 cells were treated with glucose (A) or palmitate (B). Columns, mean of three independent experiments; bars, SE. (C, D) Relative expression of miR‐1322 in various human tissues (C) and human cell lines (D). Data are means ± SEM from three independent experiments analysed in six replicates with reference to control microRNAs (U6). *P* < 0.01(**), *P* < 0.001 (***), *P* < 0.0001 (****)  NS (no significance)

To further explore the association between miR‐1322 and the metabolism of lipids and glucose, cell models for insulin resistance were induced with palmitate or high glucose cell culture media. miR‐1322 levels are significantly increased after high glucose (Figure 4A) or palmitate stimulation (Figure 4B). Although the cell viability was obviously decreased after PA (1.0 mmol/L) treatment (Figure S4), the expression of miR‐1322 was positively correlated with the concentration of PA stimulation. In addition, we found that miR‐1322 was more highly expressed in HepG2 cells (Figure 4D) compared with other cell lines, and an abundant amount of miR‐1322 was also detected in the intestine (Figure 4C), indicating that miR‐1322 could affect insulin resistance.

### 3.6 Re‐sequencing results of 3′‐UTR in ChREBP

A total of 482 Han Chinese participants were enrolled, including 169 HTG patients and 313 control participants (Table 1). Cumulatively, we identified two DNA variants in this population. Of the two variants, one was a common polymorphism (minor allele frequency >1%); the other was a rare polymorphism. No significant differences of genotype frequencies were discovered between the two groups (Table 3). We did not find significant associations between rs1051921 polymorphism and TG, TC, LDL‐C or HDL‐C (Table S5). According to the luciferase assay, significant differences of rs1051921‐C allele and T allele luciferase activity were discovered (Figure S5). However, no transcription factor or miRNA was predicted in this SNP. Only rs1051943‐A allele was found in both control and HTG patients. To eliminate racial differences, 96 Chinese Uighur ethnic participants were also included in the sequencing cohort, of which similar results were found (Table 3).

**Table 3:** Characteristics of the ChREBP 3’‐UTR variants identified by Sanger sequencing

Position^a^	SNP ID	Maj > Min	Chinese Han ethnic		Chinese Uighur ethnic(n = 96)
			Control (n = 313)	HTG (n = 169)	
ch7:73007606	rs1051943	A > G	0	0	0
ch7:73008113	rs555729695	T > C	0.0015	0	0
ch7:73007943	rs1051921	C > T	0.129	0.078^b^	0.156

Maj, major allele; Min, minor allele.

a Base pair position is based on NCBI GRCh37. All variants were in Hardy‐Weinberg equilibrium (*P* > 0.05).

b No statistical significant difference of genotype frequencies between control and HTG patient groups was discovered using chi‐squared test.

### 3.7 Conservation of 3′‐UTR in ChREBP in different species

**Figure 5:**
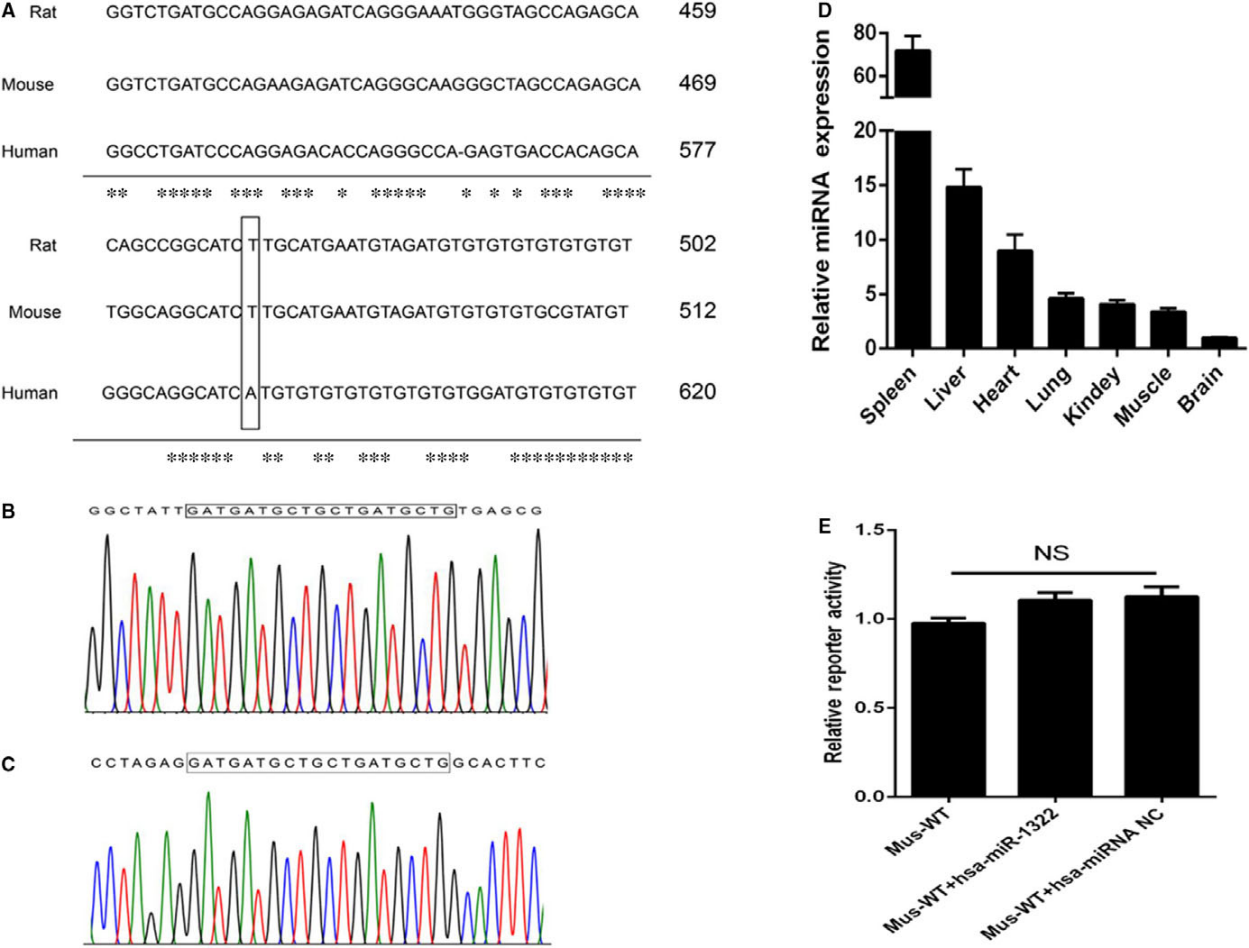
Conservation of ChREBP 3’‐UTR and miR‐1322 function in different species. A, Proximal region of rs1051943 in human, rat and mouse. Conservative bases were indicated as asterisks. B, Sequencing results of PCR product of the mouse chromosome 19 (GRCm38.p4, 29842645 to 29842663). C, Sequencing results of qPCR product of mouse liver. D, The expression level of hsa‐miR‐1322 homologue in different mouse tissues (n = 3). E, Interaction between hsa‐miR‐1322 and mouse ChREBP 3’‐UTR using reporter assay in 293T cells. Results were means of triplicate experiments with at least six replicates. NS, no significance

Sequence alignment showed that 3′‐UTR in ChREBP flanking the miR‐1322 binding site was conserved in mice and rat (Figure 5A). However, the homology allele of the rs1051943 was a T allele in both species, which could lead to the destruction of the miR‐1322 binding site. According to the existing database, the hsa‐miR‐1322 homologue was not found in both mice and rat. Therefore, we performed bioinformatics analysis with UCSC (http://genome.ucsc.edu/). The sequence of hsa‐miR‐1322 was found in chromosome 19 (GRCm38.p4, 29842645 to 29842663) (Figure 5B). Liver cDNA of mice was amplified using hsa‐miR‐1322 qPCR primers, and PCR products were then TA‐cloned into pMD 19‐T vector. Subsequent sequencing of the TA clone conformed to the same sequence of hsa‐miR‐1322 (Figure 5C), indicating the existence of a hsa‐miR‐1322 homologue in mice (Figure 5D). Luciferase activities of the mice 3′‐UTR in pMIR‐Report construct showed no significant difference after hsa‐miR‐1322 intervention in 293T cells (Figure 5E).

**Figure 6:**
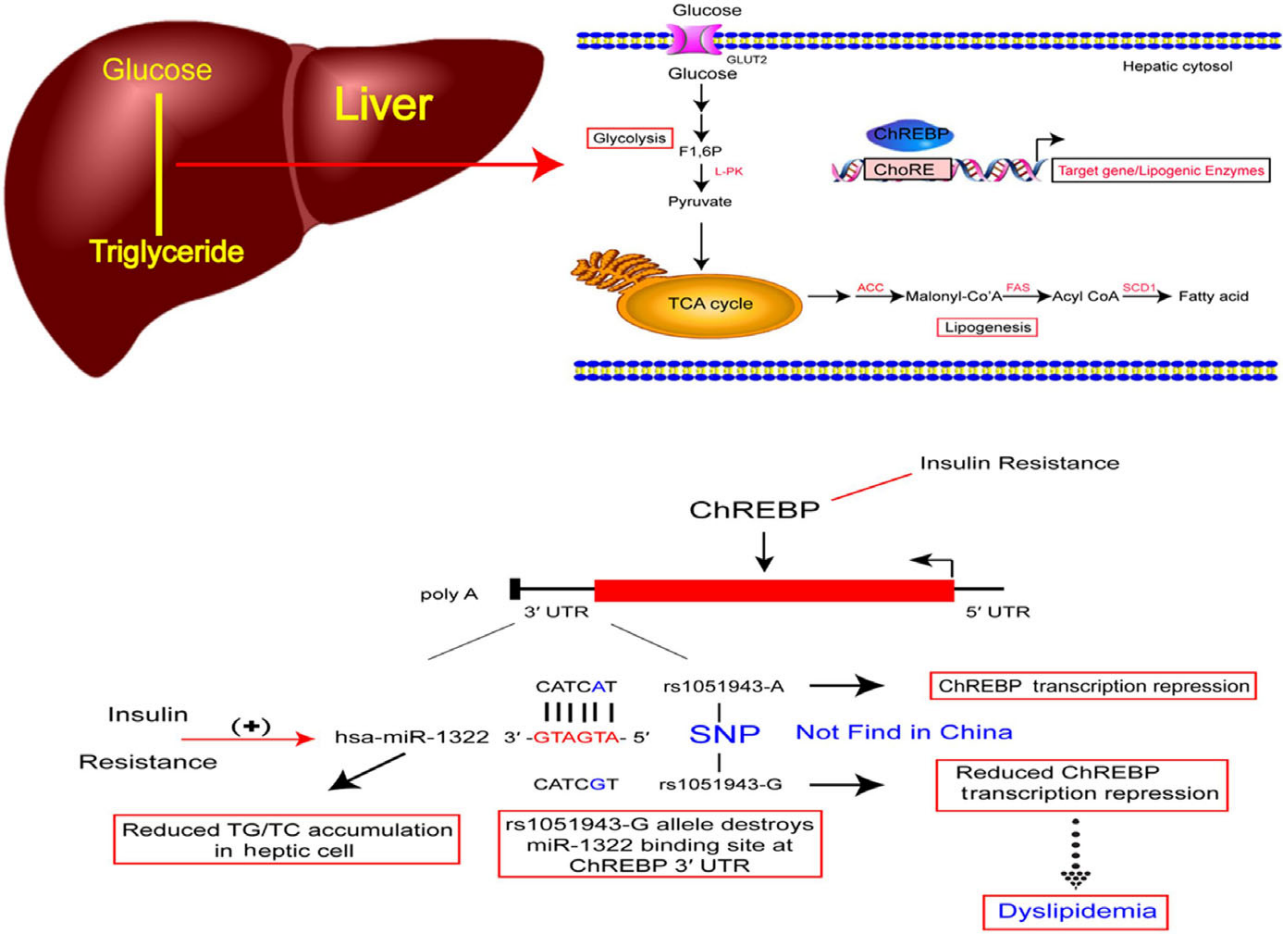
Graphical illustration of the role of miR‐1322 in ChREBP expression. The variant rs1051943 (A > G) was located in the seed binding site of miR‐1322. Through achieving miR‐1322 binding efficiency, rs1051943 A allele reduced ChREBP expression and consequently regulated the accumulation of TG/TC in HepG2 cells. Expression of miR‐1322 was induced by glucose or palmitate stimulation as a cellular insulin resistance model. The rs1051943 G allele in non‐Asian population may influence individual plasma lipid levels and the development of coronary artery disease

## 4 DISCUSSION

In this study, we identified a functional variant, the A allele of rs1051943, in the 3′‐UTR regulatory region of ChREBP suppressing ChREBP translation by facilitating miR‐1322 binding. According to our re‐sequencing results in the Chinese ethnicities, the G allele frequency of rs1051943 was different from that in European populations (MAF = 0.07). Moreover, experiments in HepG2 cells revealed that miR‐1322 participated in lipid and glucose metabolism, and the miR‐1322 expression could be regulated by glucose and palmitic acid stimulation (Figure 6).

It is clear that ChREBP plays important roles in modulating the process of glycolysis and lipogenesis.^23^ Iizuka et al reported that the ChREBP knockout mice showed reduced glycolysis and lipogenesis and exhibited insulin resistance,^24^ whereas Benhamed et al showed that the ob/ob mice with ChREBP deletion exhibited reduced lipogenesis and improved insulin sensitivity. These controversial results in different mice models were confirmed by multiple laboratories.^25^, ^26^ Recently, an American obese adolescents study revealed that the expression of ChREBP was significantly increased in the liver with high insulin resistance, which was in contrast to that in adipose tissue.^27^ Regarding the aforementioned evidence, ChREBP mechanisms in glucose and lipid metabolism warrant further exploration.

Given the important roles of ChREBP in glucose and lipid metabolism, whether genetic factors were involved requires further examination. In this study, we firstly established the interaction between the polymorphism of 3′‐UTR in ChREBP and miR‐1322. The rs1051943 A allele, not the G allele, facilitated miR‐1322‐induced down regulation of ChREBP, while inhibition of miR‐1322 could increase the expression of ChREBP. Due to the different genetic backgrounds of the hepatoma cell lines, the consistent direction of ChREBP gene and hsa‐miR‐1322 expression was not observed, as shown in Figure 4D and Figure S3. In addition, miR‐1322 could regulate the expression of ChREBP downstream target genes, but not other lipogenic genes such as the key transcription factors, LXRα and USF1. These effects could further attenuate the accumulation of TG/TC in HepG2 cells. Moreover, based on our results, the expression of miR‐1322 was positively correlated with the concentration of glucose and palmitate stimulation. To date, the function of miR‐1322 remains largely unknown. Zhang et al reported that it might act as a potential biomarker in patients with oesophageal squamous cell carcinoma.^28^ To our knowledge, this is the first report to demonstrating the association between miR‐1322 and lipids and glucose metabolism.

Several studies have demonstrated the association between ChREBP gene polymorphisms and plasma TG levels or glucose‐related traits.^8^, ^29^, ^30^ Recently, a functional coding variant in ChREBP (rs35332062) was identified in both East Asian and European populations with similar MAF (0.117 in East Asian and 0.109 in European populations, respectively).^31^ However, only the rs1051943 A allele was found in our study population, which was different from the European population (rs1051943 MAF = 0.07). Because the minor allele frequency of rs1051943 in our study was extremely low, we cannot declare the association between rs1051943 polymorphism and lipid or glucose levels as well as ChREBP gene expression level in vivo. Indeed, the relationship between rs1051943 and metabolic syndrome should be investigated in further larger populations. Meanwhile, regarding the implication of ChREBP in insulin resistance, the distribution discrepancy of rs1051943 in different ethnicities may partially explain the different susceptibility to metabolic syndrome associated diseases.^32^

Conservation analysis of the 3′‐UTR of ChREBP in different species showed that the homologous sequence of rs1051943 in mice is T allele, but not the A allele. The luciferase reporter assay confirmed our hypothesis that 3′‐UTR of ChREBP in mice might mimic the function of rs1051943 mutant G allele in human beings. miRNA sharing the same sequence of hsa‐miR‐1322 was detected in mouse tissues, and the gene locus was identified. However, based on our knowledge, the function of miR‐1322 in pathological and physiological processes of lipid metabolism is not well established, which need to be investigated in further mechanistic studies.

Our conclusions must be interpreted in the context of several limitations. Firstly, not all the variants in ChREBP gene were assessed in this study. Complete sequencing of the whole gene region of ChREBP would be necessary for systematic identification of potentially causative mutations. Another limitation was the relatively small sample size used for sequencing analysis, which could give rise to false associations by chance (type one error) or may fail to detect true differences. Therefore, it would be important to confirm these findings in prospective cohort studies in both Han Chinese populations and other ethnic groups. Furthermore, the mechanism by which miR‐1322 could regulate the levels of lipids is not fully understood and requires further elucidation. Finally, we cannot exclude the possibility that other factors may also regulate the expression of ChREBP through the miR‐1322 binding site or other regulatory region, and this needs to be investigated in future studies.

In summary, we identified a variant in the 3′‐UTR of ChREBP that functionally interacted with miR‐1322 and potentially participated in the regulation of lipid metabolism. The miR‐1322 could regulate the expression of ChREBP and further influence TG/TC production in vitro. Our findings may provide potential therapeutic strategies for metabolic syndrome.

## CONFLICT OF INTERESTS

The authors confirm that there are no conflicts of interest.

## AUTHOR CONTRIBUTIONS

Y.Z. participated in the research design, carried out the epidemiological investigation, executed the cell experiment, undertook sequencing, performed statistical analyses and drafted the manuscript. S.‐L.H participated in the research design, collected samples and performed statistical analyses. D.H. collected samples and executed the cell experiment. G.‐L.C. conceived the study, participated in the research design, collected samples and edited the final manuscript. J.‐G.J. carried out the epidemiological investigation and collected samples. All authors have read and approved the final manuscript.
