# Supplementary material for: miR‐1322 regulates ChREBP expression via binding a 3′‐UTR variant (rs1051943)
Source: J Cell Mol Med. 2018 Aug 5;22(11):5322–32. doi: 10.1111/jcmm.13805 (PMC6201350; doi:10.1111/jcmm.13805)

**miR-1322 regulates ChREBP expression via binding a 3’-UTR variant (rs1051943)**

Ying Zhang^1,2*^, Sen-Lin Hu^2*^, Dong Hu^2^, Jian-Gang Jiang^2^, Guang-Lin Cui^2^ , Xing-De Liu^1^, and Dao Wen Wang^2^

1 Department of Cardiology, Affiliated Hospital of Guizhou Medical University, Guiyang, Guizhou, 550004, China.

2 Institute of Hypertension and Department of Internal Medicine, Division of Cardiology, Tongji Hospital, Tongji Medical College, Huazhong University of Science and Technology. Hubei Province Key Laboratory of Genetics and Molecular Mechanisms of Cardiological Disorders, Wuhan 430030, China.

*Y. Zhang and S.L. Hu contributed equally to this work.

Corresponding Authors:

Dao Wen Wang, MD, PhD

Division of Cardiology, Department of Internal Medicine

Tongji Hospital, Tongji Medical College

Huazhong University of Science & Technology

Hubei Province Key Laboratory of Genetics and Molecular Mechanisms of Cardiological Disorders

Wuhan 430030, People’s Rep. of China

Tel. & Fax: 86-27-8366-3280

Email: dwwang@tjh.tjmu.edu.cn

Xing-De Liu, MD, PhD

Department of Cardiology

Affiliated Hospital of Guizhou Medical University

Guiyang, Guizhou, 550004, China

Email address: lxd@gmc.edu.cn

Running title: miR-1322 regulates ChREBP expression

| **Supplementary Table 1. Sequences of miRNA-1322 mimics and inhibitors.** | |
| --- | --- |
| **miR ID** | **Sequence** |
| hsa-miR-1322 mimics | 5'-GAUGAUGCUGCUGAUGCUG-3'  5'-CAGCAUCAGCAGCAUCAUC-3' |
| hsa-miR-1322 inhibitor |

Supplementary Table 2. Baseline characteristics of the Chinese Uighur ethnic group.

|  |  |
| --- | --- |
| **Characteristics** | **Uighur ethnic （n=96）** |
|
| Age,years | 56.5±9.8 |
| Men，% | 46.8 |
| SBP，mm Hg | 124.4 ± 7.5 |
| DBP，mm Hg | 76.6 ± 4.7 |
| BMI，kg/m2 | 28.40 ± 3.38 |
| Hypertension | 0 |
| Type 2 diabetes | 0 |

**Supplementary Table 3.** Primer sequences for real-time PCR.

| **Gene Name** | **Forward Primer Sequence** | **Reverse Primer Sequence** |
| --- | --- | --- |
| ChREBPα | AGTGCTTGAGCCTGGCCTAC | TTGTTCAGGCGGATCTTGTC |
| ChREBPβ | AGCGGATTCCAGGTGAGG | TTGTTCAGGCGGATCTTGTC |
| PKLR | GTGGACATCGTCTTTGCCT | TCTTGATGCCGTGTCCTTC |
| FASN | CGCTCGGCATGGCTATCT | CTCGTTGAAGAACGCATCCA |
| ACC | GGATGGTGTTCACTCGGTAATAG | GGGTGATATGTGCTGCGTCAT |
| SCD1 | TACAAGAGTGGCTGAGTTTGG | GCATCCTGGTAGCATTATTCA |
| 18sRNA | CGAACGTCTGCCCTATCAACTT | ACCCGTGGTCACCATGGT |
| SREBP1 | CGGAACCATCTTGGCAACA | GCCGGTTGATAGGCAGCTT |
| LXRa | AGAAGAACAGATCCGCCTGAAG | GGCAAGGATGTGGCATGAG |
| USF1 | CACCACGGATTAGAGGTCG | CACCACGGATTAGAGGTCG |

**Supplementary Table 4. List of the** miRNAs predicted in **ChREBP 3′ UTR region.**

| **miRNA Name** | **Start site^a^** | | **End site^a^** | **Website** |
| --- | --- | --- | --- | --- |
| miR-497 | 2 | 23 | | microrna.org |
| miR-15a | 1 | 23 | | microrna.org |
| miR-15b | 1 | 23 | | microrna.org |
| miR-16 | 1 | 23 | | microrna.org |
| miR-195 | 2 | 23 | | microrna.org |
| miR-214 | 1 | 20 | | microrna.org |
| miR-424 | 1 | 22 | | microrna.org |
| miR-490-3p | 122 | 146 | | microrna.org |
| miR-22 | 303 | 324 | | microrna.org |
| miR-542-3p | 377 | 399 | | microrna.org |
| miR-873 | 424 | 465 | | microrna.org |
| miR-326 | 480 | 500 | | microrna.org |
| miR-330-5p | 478 | 500 | | microrna.org |
| miR-370 | 460 | 582 | | microrna.org |
| miR-874 | 564 | 586 | | microrna.org |
| miR-129-5p | 639 | 660 | | microrna.org |
| miR-336-3p | 650 | 670 | | microrna.org |
| ^a^Start and end site were numbered relative to the stop codon based on NCBI GRCh37, NM 032951 as reference. | | | | |

**Supplementary Table 5.Association of rs1051921 genotype with plasma TG, TC, LDL-C or HDL-C levels adjusted for sex and age.**

| **The general population** | **Rs1051921** | | | **Beta(SE)** | **Trend p Value** |
| --- | --- | --- | --- | --- | --- |
|  | **CC（n）** | **CT（n）** | **TT（n）** |  |  |
| TG | 238 | 69 | 6 | -0.077(0.052) | 0.135 |
| TC | 238 | 69 | 6 | -0.004(0.107) | 0.968 |
| HDL | 238 | 69 | 6 | 0.068(0.044) | 0.125 |
| LDL | 238 | 69 | 6 | -0.057(0.085) | 0.499 |

**Supplementary Figure 1. Transfection efficiency of different cell lines. Cy3-labeled transfection control were transfected into HepG2 cells (A) and 293T cells (B). Fluorescent microscopy, 200×.**

**
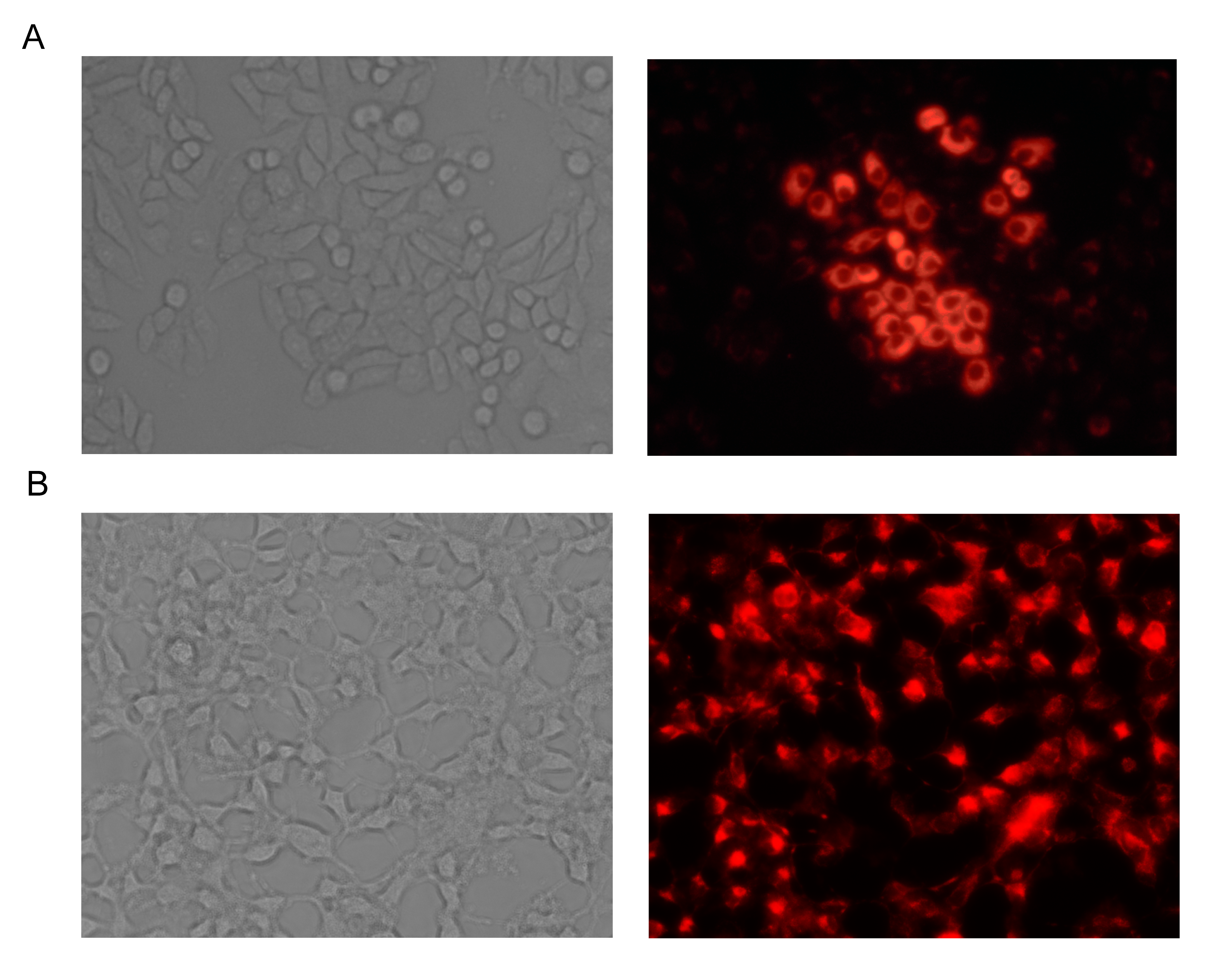
**

**Supplementary Figure 2. S**equencing results of the **ChREBP 3′ UTR flanking rs1051943 in different hepatic cell lines. a, Hep1; b, Hep3B; c, SMMC-7721; d, L02. The rs1051943 locus was indicated with black arrows.**

**
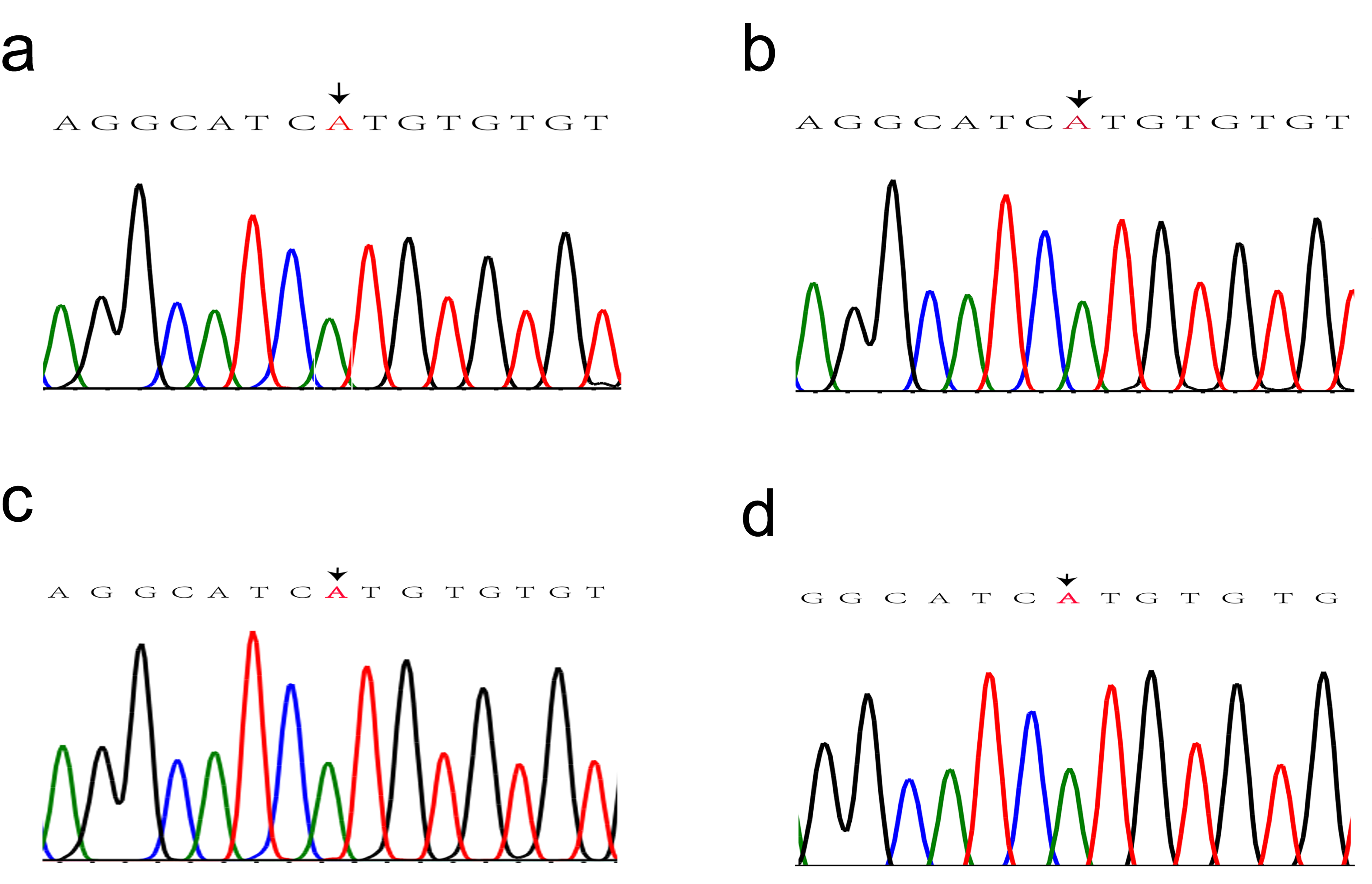
**

**Supplementary Figure 3. ChREBP gene expression in different hepatic cell lines.**


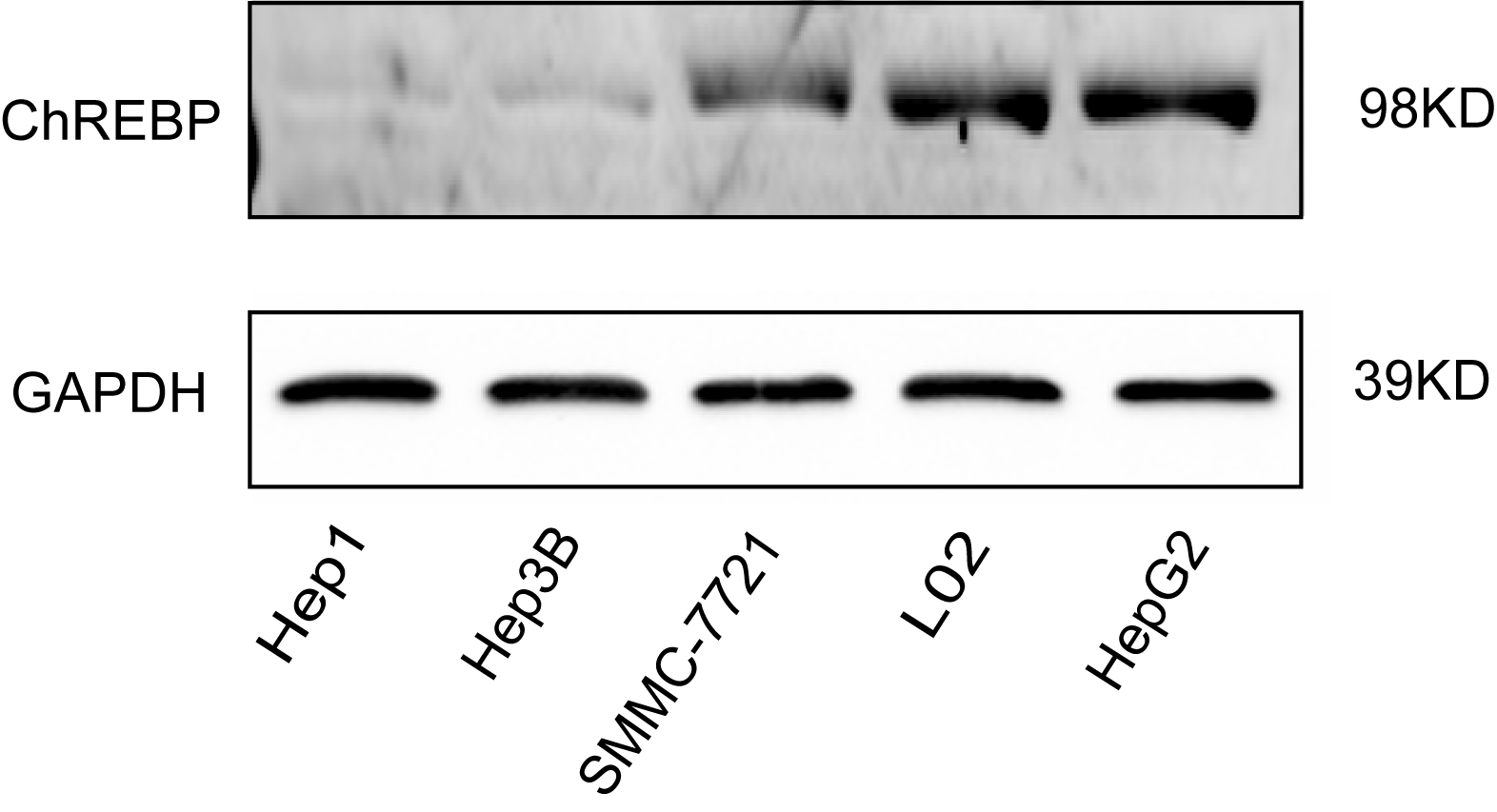


**Supplementary Figure 4**. **Effect of different concentrations of palmitate and glucose stimulation on cell viability. A, HepG2 cells were treated with different concentrations of PA (0.125–1.0mM) for 12–24h. B, HepG2 cells were treated with different concentrations of glucose (5.5–25mM) for 12–24h. All values are mean ± SEM of three independent experiments. p <0.05 (*), p <0.01 (**), P<0.0001 (****), compared with control group.**

**
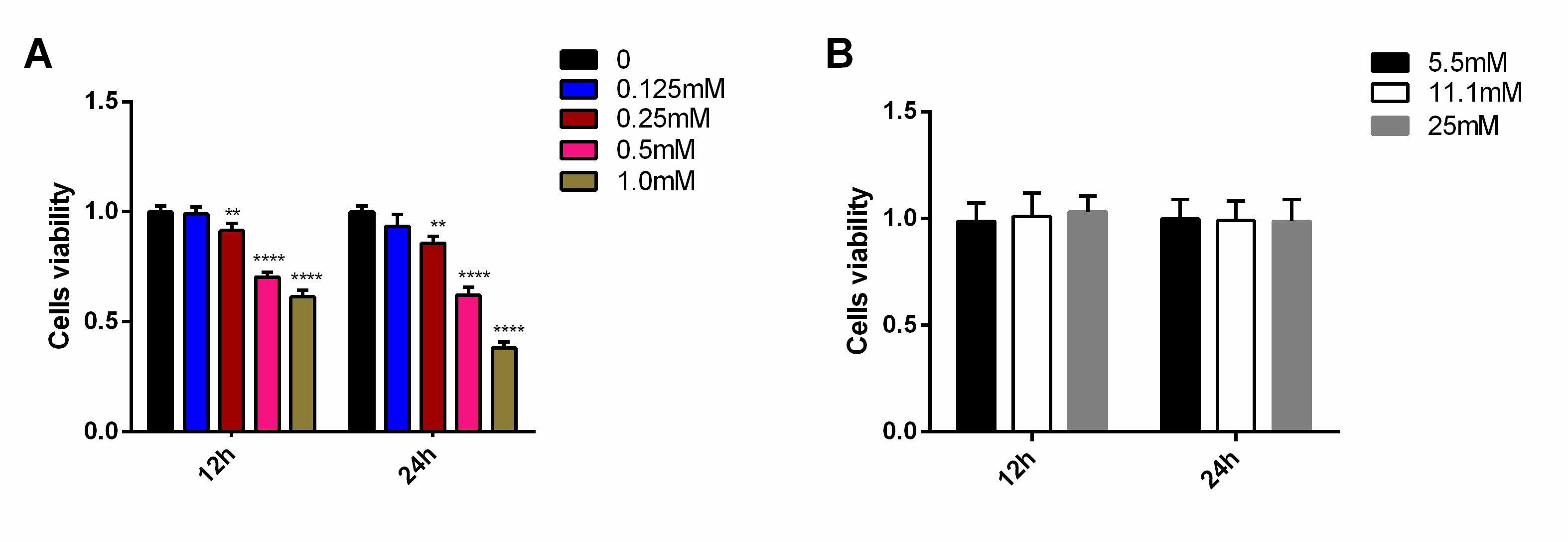
**

**Supplementary Figure 5**. **Effect of rs1051921 C/T alleles on ChREBP 3’ UTR constructs activity in HepG2 cells (A) and 293T cells (B). ChREBP 3’ UTR Reporter activty is expressed relative to the co-transfected Renilla luciferase activity. Three independent experiments containing six replicates were performed in each cell line. For each comparison, mutant construct was compared with the wild-type construct. All values are mean ± SEM of three independent experiments. p <0.05 (*), p <0.01 (**), p <0.001 (***), P<0.0001 (****), NS (no significance).**


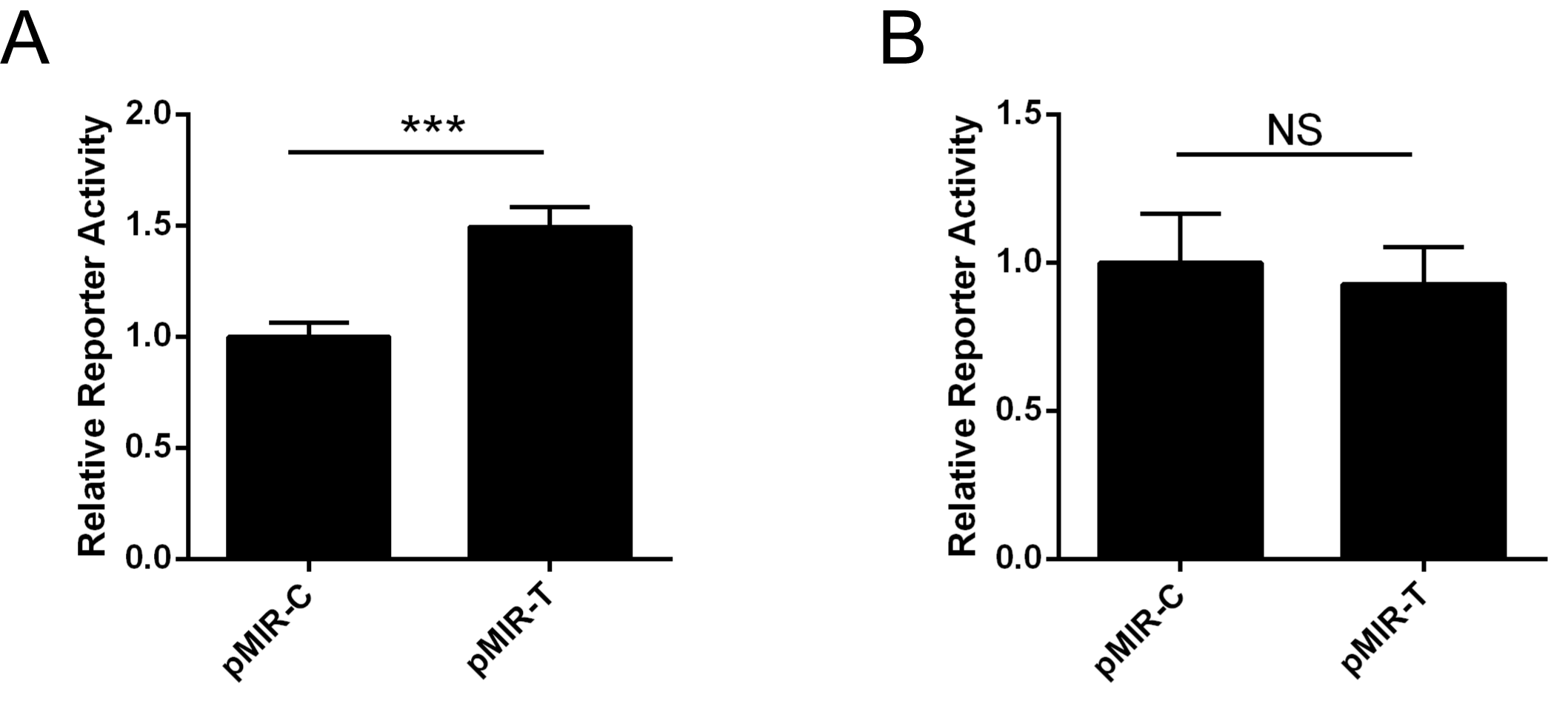

Supplement: Supplementary file 1 [file JCMM-22-5322-s001.doc]
